# Dynamic processes of the first NHC-substituted rhenium heptahydrides [ReH_7_(NHC)_2_]

**DOI:** 10.1039/d5dt01655j

**Published:** 2025-08-04

**Authors:** G. Grieco, A. Pierini, M. Pierini

**Affiliations:** a University of Zurich, Department of Chemistry Winterthurerstrasse 190 CH-8057 Zurich Switzerland pgag.2021@gmail.com +4144 635 68 02; b Sapienza University of Rome, Department of Chemistry Piazzale Aldo Moro 5 00185 Roma RM Italy adriano.pierini@uniroma1.it; c Sapienza University of Rome, Department of Drug Chemistry and Technology Piazzale Aldo Moro 5 00185 Roma RM Italy

## Abstract

In this paper are presented possible geometries and fluxional mechanisms of three [ReH_7_(NHC)_2_] complexes (NHC = IMes (1) (1,3-bis(2,4,6-trimethyl phenyl) imidazol-2-ylidene), IMesCl_2_ (2) (4,5-dichloro-1,3-dihydro-1,3-bis(2,4,6-trimethylphenyl)-2*H*-imidazol-2-ylidene), BIMes (3) (1,3-bis(2,4,6-trimethylphenyl)-benzo imidazole ylidene)). Using DFT calculations, plausible fluxional processes were proposed, and a new process named “η^2^-quadratic twist” has been identified. For 2 the intermediate complex [Re(H)_5_(η^2^-H_2_)(IMesCl_2_)_2_] proved to be more energetically accessible at RT than those of the complexes 1 ([Re(H)_5_(η^2^-H_2_)(IMes)_2_]) and 3 ([Re(H)_5_(η^2^-H_2_)(BIMes)_2_]). Two different fluxional mechanisms have been proposed: the first one for 1 and 3, and the second one for 2. The mechanism for complex 2 implies the C–H activation of the hydrogens of the *o*-CH_3_ group of the IMesCl_2_ ligands.

## Abstract 2

Possible geometries and fluxional mechanisms of three [ReH_7_(NHC)_2_] complexes (NHC = IMes (1), IMesCl_2_ (2), and BIMes (3)) are presented. A new process named “η_2_-quadratic twist” has been identified. The complexes presented can perform C–H activation.

## Graphical abstract

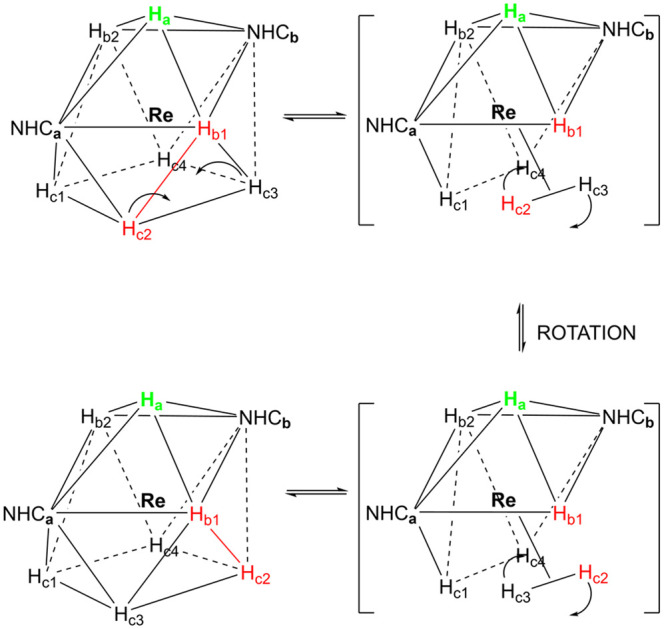

## Introduction

### Transition metal polyhydrides

**Fig. 1:**
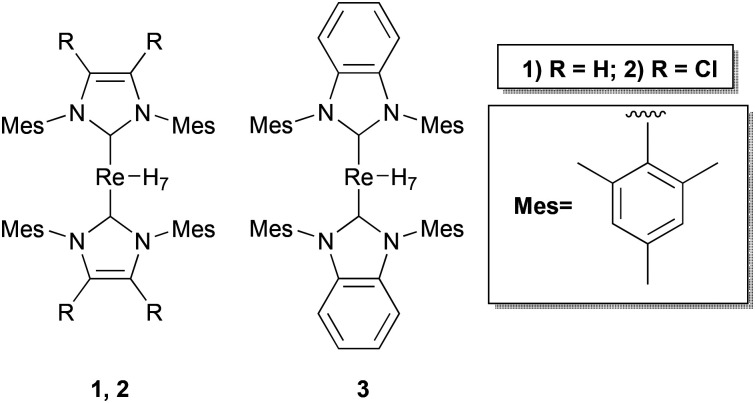
Re–NHC heptahydrides 1–3 considered in this work.

Hydride transition metal complexes are of extreme importance in organometallic chemistry and inorganic chemistry, and are becoming increasingly more important in fields such as materials chemistry and engineering. Exemplary uses of metal-hydrides can be found in different fields of engineering, such as thermal energy storage,^1^ actuation in rehabilitation,^2^ optics,^3^ hydrolysis batteries,^4^ and hydride batteries.^5^ For instance, thin film forms of Mg-alloy hydrides showed interesting electrochromic properties depending on their content of H.^6^ In fact, while the metal reflects the light, its metal–hydride complex shows a transparent state. The modern era of metal-polyhydrides probably started with the complete characterization of the first polyhydride [K_2_ReH_9_] (potassium nonahydridorhenate) made by Ginsberg in 1964.^7^ However, to understand the structural properties of the polyhydridic complexes and to rationalise their chemical behaviour and reactivity, we must also determine their internal structure. The aforementioned properties depend on factors like the type of metal, the oxidation state (OS) of the latter, and the type of the ligands bound to the metal. The σ-donor or π-acceptor abilities of the ligands are able to influence the electronic properties of the metal centre, dictating the way in which the hydrides would behave. The bulkiness of the ligands can significantly influence the geometry of the complex in both the solution and solid state. In fact, as shown by Luo and Crabtree^8^ first, and more recently by Grieco and Blacque,^9^ the steric hindrance of the ligands is able to affect the internal equilibrium of dihydrogen\dihydride (H_2_\2H^−^), leading to intermediates with a H_2_ coordinated η^2^ (3 centre–2 electron-non-classical bond). The polyhydrides proved to be fluxional complexes,^10–14^ and the active process that continuously changes the position of each hydride around the metal centre without breaking any bond, is called a polytopal rearrangement.^15^ It is of great importance to study the isomerism since the way in which atoms are arranged in whatever compound has a deep influence on both its chemical and physical properties.^16^ Most of the time, internal dynamic processes take place, giving rise to many intermediate species that would differ from the one determined by means of crystallographic methods. In this work we will consider three Re complexes of general formula [ReH_7_(NHC)_2_] with NHC = IMes (1) (1,3-bis(2,4,6-trimethyl phenyl) imidazol-2-ylidene), IMesCl_2_ (2) (4,5-dichloro-1,3-dihydro-1,3-bis(2,4,6-trimethylphenyl)-2*H*-imidazol-2-ylidene), and BIMes (3) (1,3-bis(2,4,6-trimethylphenyl)-benzo imidazole ylidene), which have been synthesized and characterized in a previous work (Fig. 1).^9^

**Scheme 1:**
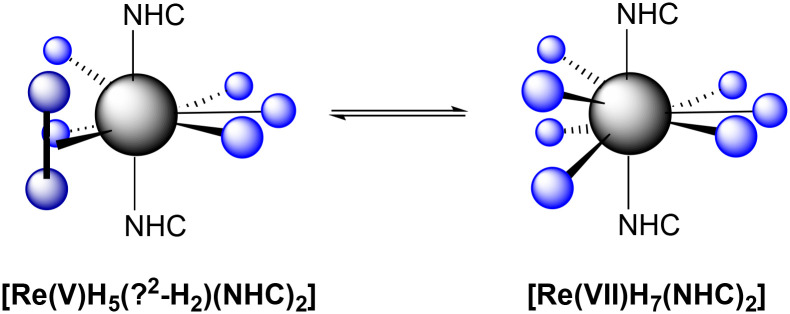
Re NHC–heptahydrides 1–3 interconvert rapidly from Re(vii) to Re(v) and *vice versa*. NHCs = IMes (1), IMesCl_2_ (2) and BIMes (3).

The complexes 1–3, as we will see later, are actually present in solution as two species rapidly interconverting into each other, *i.e.* [Re(vii)H_7_(NHC)_2_] ↔ [Re(v)H_5_(η^2^-H_2_)(NHC)_2_] (Scheme 1). The two Re complexes in equilibrium have seven hydrides that are held together through different bonds. Therefore, they can be considered constitutional isomers interconverting into each other.

### Possible structural alternatives for 1–3

**Fig. 2:**
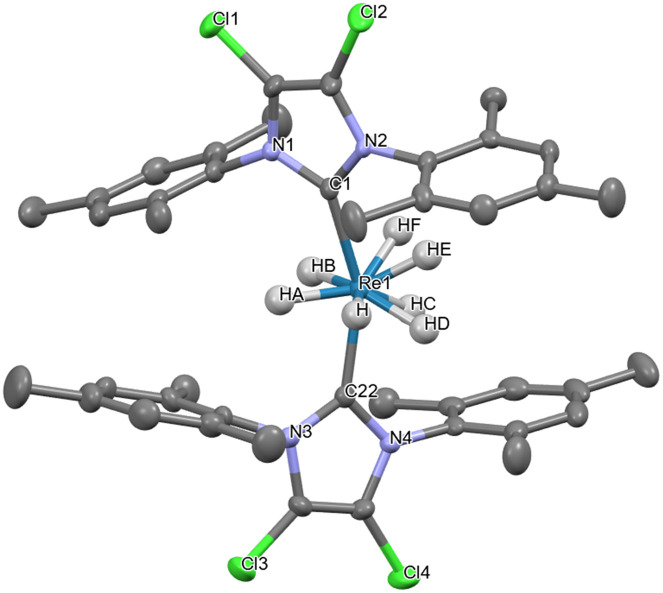
The crystal structure of complex 2 revealed a distorted CSA geometry.^9^ Selected distances (Å): *d*_H–HA_ = 1.929; *d*_HA–HB_ = 1.752; *d*_HF–HD_ = 1.418; *d*_HF–HE_ = 1.634; *d*_HE–HC_ = 1.565; *d*_HC–HD_ = 1.824; *d*_H–HD_ = 1.614; *d*_HF–H_ = 1.998; *d*_HB–HC_ = 2.047; *d*_HF–HB_ = 1.880.

Despite the classical heptahydride structure adopted in the solid state by 1–3, the complexes may assume other geometries in solution, because of the continuous scrambling of the hydrides and the possible formation of η^2^-H_2_. In group 7 besides Re, also Tc was able to coordinate nine hydrides, while for Mn, Singh and Gupta, through DFT calculations, argue that the unknown BaMnH_9_ material, containing the [MnH_9_]^2−^ ion, may prove to be stable.^17,18^ The complexes K_2_ReH_9_^19,20^ and K_2_TcH_9_^21^ are examples of homoleptic complexes with a tricapped trigonal prismatic (TTP) geometry.^22^ The crystal structure of the complex [ReH_7_(IMesCl_2_)_2_] (2) (Fig. 2) was revealed fortuitously by means of X-ray.^9^ The geometry of complex 2 in the solid state can be described as capped square antiprismatic (CSA), with hydrides H_C_, H_D_, H_E_, and H_F_ representing the vertices of the square base of the antiprism.^9^ The hydrides H and H_B_ along with the carbon of the carbenes C_1_ and C_22_ represent the vertex of a distorted square, while H_A_ is the cap on the latter. Connecting all the vertices just mentioned we could draw the distorted CSA geometry cited above. It must be said that the difference between a TTP geometry or a CSA one is subtle, particularly because the complexes exhibit geometries with a certain degree of distortion. In the literature several publications about complexes are present that have an internal equilibrium in which they pass from a TTP geometry into a CSA one and *vice versa*.

For instance, Spirlet and co-workers reported that in the structure of the barium tetrakis(oxalato)uranate(iv) octahydrate, complex (Ba_2_U(C_2_O_4_)_4_·8H_2_O), the metal centre (U) is nona-coordinated since there are four oxalate groups that act as bidentate ligands and a single molecule of H_2_O that coordinate through its O.^23^ The authors described the geometry of the Ba_2_U(C_2_O_4_)_4_·8H_2_O complex like something between the TTP and the CSA one. Another case similar to the latter is the one studied by Zych and co-workers in which the U of the complex ((NH_4_)[UBr_2_(CH_3_CN)_2_(H_2_O)_5_]Br_2_) showed a coordination geometry that is intermediate between a TTP and a CSA.^24^ A more representative case, is the one described by Kraka and Tao, who with the help of DFT calculations, found a very low energy barrier (approximately 0.3 kcal mol^−1^) for the transition of (ReH_9_)^2−^ from its TTP geometry to a higher-energy CSA geometry.^13^ As pointed out by Naik and Moehring, this transformation may occur in many Re(vii) complexes, likely making their dynamic behaviour too rapid to allow the acquisition of slow-exchange NMR spectra in solution.^12^ The problem in assigning the proper geometry in complexes containing hydrides is mostly due to several fluxional transformations that may take place inside them. Examples of these processes are: the polytopal rearrangements,^13–15^ H-atom exchange in stable *cis*-complexes in which the process 2H^−^ ↔ η^2^-H_2_ occurs,^25^ orbital-like motion of hydride ligands around low-coordinate metal centres containing NHCs,^26^ 2H^−^ ↔ η^2^-H_2_ ligand transformations,^27^ and the 2H^−^ ↔ η^2^-H_2_ exchange involved in the C–H activation (“↔” here indicates an equilibrium and not a resonance).^9^ The last two transformation mentioned require the metal centre (M) to possess the ability to undergo readily and reversibly to a M(*n*)\M(*n* + II) variation of its OS.

### Charge distribution analysis

The complexes 1–3 are stable 18 electron complexes with seven hydrides giving just bonding orbitals with empty non-bonding orbitals, and two NHC ligands per complex, which also give rise to empty non-bonding orbitals. In fact, in this case, Re is in a high OS in each of the complexes considered, and that because the NHCs are not acting as π-acceptors not allowing any significant back donation that could fill their non-bonding orbitals. Besides the electronic considerations, to understand the influence of the different NHCs of the complexes 1–3 on the internal fluxional mechanisms that involve the seven hydrides, we need to consider both the different donor abilities, and the steric hindrance of the NHCs involved. The impact of the steric factor on the 2H^−^ ↔ η^2^-H_2_ equilibrium is going to be explained later in the article. The sigma-donor ability of the three different NHCs considered here is: IMes > BIMes > IMesCl_2_.^28,29^ The higher the electron donation of the NHCs to Re, the greater the π-back donation of Re into the σ* orbital of H_2_, that would trigger the cleavage of η^2^-H_2_ and consequently the dihydride formation. Instead, more electron-deficient NHCs, as the two IMesCl_2_ of 2 would render the Re electron poor, making the latter less prone to a π-back donation into the σ* orbital of H_2_ thus fostering the formation of H_2_ over 2H^−^. In order to evaluate the donor ability of the tree NHC ligands, we performed density functional theory (DFT) calculations with the B3LYP exchange–correlation functional and def2-TZVP basis set on the [Re(vii)H_7_(NHC)_2_] structures 1–3 (geometry optimizations are referred to in the section DFT calculations). The extent of charge transfer occurring from the ligands to the Re centre (Table 1) was estimated through Extended Charge Decomposition Analysis^30^ (ECDA), as implemented in the Multiwfn 3.8 code distribution,^31^ and it confirmed their donor ability as reported in the literature.^28,29^ Both IMes and BIMes, being average electron donors, favour the transformation of H_2_ in 2H^−^ in a fast equilibrium (2H^−^ ↔ η^2^-H_2_), while IMesCl_2_, being a weaker electron donor, favours the formation of η^2^-H_2_.

**Table 1:** Charge transfer of NHCs in [ReH_7_(NHC)_2_] complexes estimated through ECDA. The numbers shown represent the fraction of electronic charge that is transferred from the two NHCs to Re through the bonds

NHC	Charge transfer
IMes	0.898
BIMes	0.852
IMesCl_2_	0.834

**Fig. 3:**
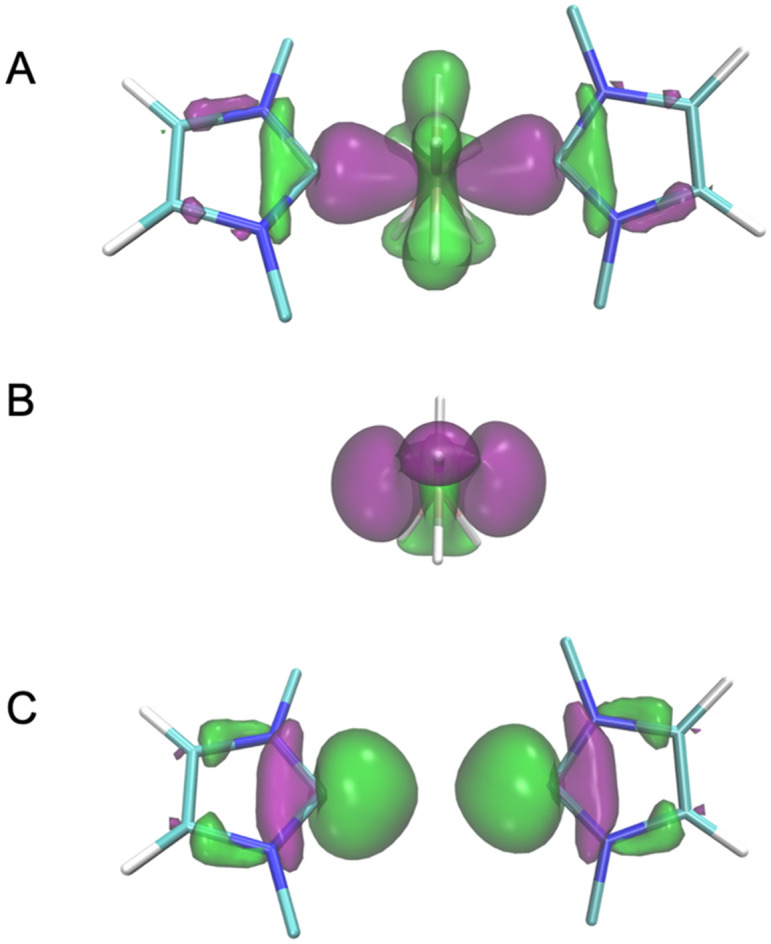
For NHC = IMes, representation of: the MO with the largest contribution to the NHC donation to ReH_7_ (A), corresponding unperturbed acceptor MO of ReH_7_ (B), corresponding unperturbed donor MO of NHC (C). For clarity, mesityl substituents are not shown in the plots.

Through ECDA, the molecular orbitals (MOs) with the largest contribution to the ligand-to-metal donation (Fig. 3A) were identified for 1–3. These MOs in the complex are mainly formed by one occupied donor MO in the ligand (Fig. 3B) and one empty acceptor MO in the ReH_7_ polyhydride (Fig. 3C). The energy of the donor MO in the unperturbed NHC ligand (Table 2) follows the same aforesaid order IMes > BIMes > IMesCl_2_, which reflects the proclivity of the respective NHC towards sigma-donation. The values of the HOMOs of the NHCs also confirm that the IMesCl_2_, being the weakest donor, has the lowest value of the HOMO (−5.52 eV).

**Table 2:** Energy of the unperturbed MOs in the NHC ligand: MO with the largest contribution to the sigma-donation and relative HOMO

NHC	Donor MO (eV)	HOMO (eV)
IMes	−5.41	−5.04
BIMes	−5.64	−5.25
IMesCl_2_	−5.88	−5.52

### Polytopal rearrangements and steric hindrance

As stated before, the ligands of a polyhydride form a polyhedron centered on the metal, which can undergo stereoisomerization resulting in the interconversion of distinct or identical spatial arrangements of the ligands (the latter are the vertices of the polyhedron) around the central atom. A well-documented example of these polytopal rearrangement of a Re polyhydride is given by Crabtree and co-workers who synthesized Re phosphine heptahydride complexes of the general formula ReH_7_L_2_ (L_2_ = a chelating bidentate phosphine) with conformationally restrictive chelating diphosphines used to slow down the fluxional processes and help the ^1^H-NMR *T*_1_ analysis used to clarify the structures of the polyhydride complexes and the nature of the metal-hydride bond.^32^ In an earlier work, Miller and Caulton found a correlation between the diphosphine chelate ring size “*n*” of complexes [Ir{Ph_2_P(CH_2_)_*n*_PPh_2_}_2_CO]^+^ and the magnitude of the barrier of intramolecular rearrangement in the trigonal–bipyramidal and octahedral complexes.^33^ The authors showed how the intramolecular rearrangement barriers decrease when the ring size of the bidentate phosphines decreases. All the [ReH_7_L_2_] containing phosphines, synthesized by the co-workers of Crabtree gave evidence of a TTP geometry.^32^ Another factor that may influence the dynamic molecular processes (*i.e.*, the fluxionality in a complex) in polyhydride Re complexes containing phosphines is the denticity of the ligands. In fact, bidentate phosphines act, making the metal centre resistant to H_2_ loss and making it unreactive towards coordinating compounds such as pyridine, when compared with the monodentate ones. The complexes with monodentate ligands are not only capable of losing H_2_, but have also shown to undergo reactions such as C–H activation^32^ It must be noted that ligands such as carbenes are high field ligands (strong σ-donor and π-acceptor properties),^34,35^ and they not only can stabilize the Re–H bond, but we can also expect that, being carbenes such as IMes and IPr even more sterically demanding than phosphines,^36^ they would act hindering the intramolecular rearrangements inside the polyhydride complexes more than the bulkiest phosphine could do. Because of the fluxionality, the complexes aforementioned have different structures that are thermally accessible within temperatures close to room temperature. Some of these structures may even have the same free energy content because of their similarity in structure and bonding.^37^ This exchange of ligands may take place by means of a rotation or a torsional motion about an axis or a pseudo-axis that does not require a bond cleavage as, for instance, in the well-known Rây–Dutt twist^38^ (or rhombic twist) and the Bailar twist^39^ (or trigonal twist). These mechanisms are really similar to each other and were reported for the first time as taking place in complexes containing chelate ligands. However, they can also take place in complexes with monodentate ligands such as in [Ru(CO)H_2_(PPh_3_)_3_].^38^ It is worth noting that it has been shown that the Rây–Dutt twist is a special case of the general Bailar twist.^40^

### Possible internal fluxional processes

**Fig. 4:**
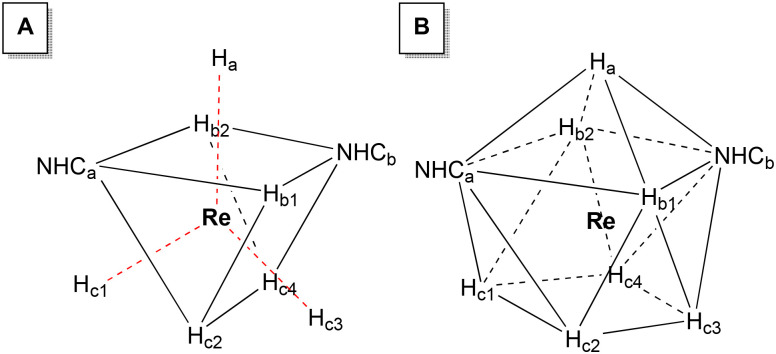
The polyhedrons showing the possible ground state geometries of 1–3 in which they may interconvert: distorted monocapped square antiprismatic (A), distorted TTP (B). Both belong to the point group *C*_2v_.

The complexes 1–3 are nine-coordinated and they could assume both pseudo-TTP and pseudo-CSA geometries (Fig. 4). The problem is that we use geometries of ideal polyhedra to describe distorted crystal structures. Therefore, it is difficult to clearly see just one well-defined geometry. Furthermore, since the hydrides are in constant motion, the TTP and CSA geometries can easily interconvert through slight movements of a few hydrides as stated before. The geometrical changes are so small that in cases of symmetry lowering, due to two different types of ligands in the heteroleptic complexes, the two basic structure types can hardly be distinguished.^42,43^ The interconversion of both structures needs no permutation of ligands; rather a small digression of two ligands occurs from their positions in the ideal coordination polygon. Homoleptic complexes such as [ReH_9_]^2−^ usually exhibit either a TTP geometry or a CSA one. This indicates that probably the last-mentioned geometries are similar in energy content since they can ensure positions in which the atoms experience the minimum repulsion between each other. The polyhydrides 1–3 are supposed to be isostructural with the [ReH_9_]^2−^ anion.^4^ In our case, we place Re in the centre of a trigonal prism, the vertexes of which are formed by four of the seven hydrides available (H_b1_, H_b2_, H_c2_, and H_c4_) and by the two NHCs (Fig. 3 A), while the other three hydrides (H_a_, H_c1_ and H_c3_) form the caps of the tricapped trigonal prism.

It would also be possible to describe the geometry of the complexes 1–3 instead as CSA. Placing Re in the centre of a monocapped square antiprism, the square at the base of the latter can be built connecting the four H_C_, while the pyramid on the top of the antiprism can be built connecting the hydrides: H_a_, H_b1_, and H_b2_ and the two NHCs (Fig. 3B). The mono capped square antiprism in solution has the chance to exhibit alternative geometries, that allow the maximum number of bonding interactions between the AOs of the hydrides and the ones of the metal. These geometries are the ones with the minimum energy content compared to all the possible geometries, and therefore they are usually found in the solid state for Re(vii) polyhydride complexes.^12^ In the literature, several articles are present describing the fluxional processes that may take place inside metal hydride complexes, such as: the diamond-square-diamond (dsd) process,^41,42^ the trigonal twist,^39^ the circle dance and three-arm turnstile mechanisms,^13^ and orbital like motion.^26^ The mechanisms that may take place in 1–3 must involve the presence of the intermediate [Re(H)_5_η^2^-H_2_(NHC)_2_] that rapidly converts into the isomer with seven hydrides, as showed by Grieco and Blacque.^9^

### Presence of η^2^-H_2_

**Scheme 2:**
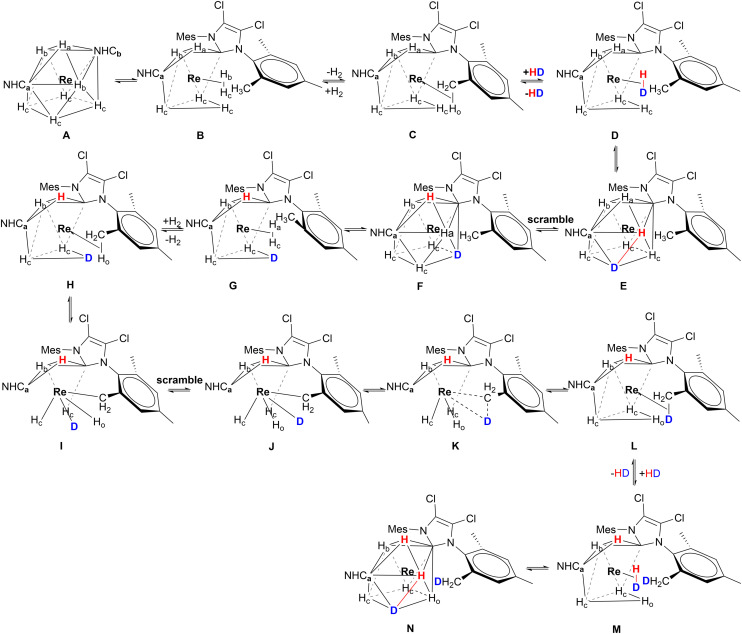
The fluxional mechanism that includes the C–H activation process which takes place in complex 2 at RT. This mechanism involves the exhaustive deuteration of all the *ortho*-methyl groups of both IMesCl_2_ in the presence of HD. The second IMesCl_2_ is not shown for clarity.

If we consider the distances between the seven hydrides present in the solid-state structure of complex 2 (Fig. 2), we find that the shortest distance measured is H_F_⋯H_D_ (1.418 Å), the second shortest distance is H_E_⋯H_C_ (1.565 Å), and both fall in the range of 0.74–1.6 Å that defines the non-classical hydrides. However, also the distances of both H⋯H_D_ (1.614 Å) and H_F_⋯H (1.614 Å) could be considered just slightly out of the range of the non-classical hydrides. The distances of both H_F_⋯H_D_ and H_E_⋯H_C_ suggest a significant H⋯H bonding interaction, far from the Kubas-type complexes (H_2_, *d*_H–H_ = 0.8–1.0 Å); however they still fall within the range of compressed hydride distances (1.1–1.6 Å).^44^ The compression of the hydrides originates from the bulky NHCs. As mentioned before, Miller and Caulton found a correlation between the steric hindrance of the ligands of a complex and the magnitude of the barrier of intramolecular rearrangement,^33^ and Crabtree showed that this phenomenon could be used to influence the H_2_/2H^−^ internal rearrangement in polyhydride complexes.^32^ Therefore, the steric hindrance of the mesityls of the NHCs of the complexes 1–3, plays an important role in the 2H^−^ ↔ η^2^-H_2_ internal fluxionality. The ^1^H-NMR spectrum of the complexes 1–3 did not reveal any change at lower temperature.^9^ For instance, the ^1^H-NMR of 1 at 193 K shows nothing more than a slight shift of the signals of 1 or 2 ppm toward a higher field when compared with the spectrum at 293 K (Fig. S1). Moreover, also the *T*_1 min_ experiments carried out for hours at temperatures reaching 193 K, did not reveal any change in the NMR spectra of the complexes 1–3. However, the H/D exchange experiments made on the complexes 1–3 provided evidence that all the complexes were able to form H_2_ and then exchange it with HD (Fig. S2–S4). This is particularly evident for complex 2, which, in addition to the hydride–deuteride exchange process, shows clear signs of deuteration at the methyl groups in the *ortho* position (*o*-CH_3_) of the mesityl rings on its NHC ligands (Fig. S5). Considering this, we hypothesized that the exchange took place through a C–H activation process involving an agostic interaction (Scheme 2). The CSA geometry (Scheme 2A) has been chosen because it is the most similar to the geometry found in the solid state (Fig. 2); however, in solution all the complexes may also adopt a TTP geometry. The processes considered here will be analysed later in the article with the help of DFT calculations. For 2 the process begins when two hydrides connect forming a H_2_ molecule coordinated η^2^ (Scheme 2B), that then leaves the complex freeing a coordination site. Then, the *o*-CH_3_ coordinates to Re connecting through a C–H → Re agostic interaction (3-center-2-electron M–H–C bond) (Scheme 2C). The agostic interaction ends when a molecule of HD coordinates to Re (Scheme 2D). The HD molecule splits and the deuterium mixes with the other hydrides already bound to the Re due to the fluxionality processes (Scheme 2E–F). Subsequently, a new molecule of H_2_ forms (Scheme 2G), and then leaves (Scheme 2H), while a new agostic interaction happens. During the agostic interaction, the proton bound to the *o*-CH_3_ group of the mesityl substituent takes part in the scrambling together with the other hydrides (Scheme 2I). D^−^ scrambles by swapping its position with the other H^−^, ending up in the position originally occupied by the H_o_ bound to the *o*-CH_3_ group (Scheme 2J and K). Eventually, D binds the *o*-CH_2_ forming *o*-CH_2_D (Scheme 2L) and a molecule of HD coordinates to Re (Scheme 2M), becoming part of the complex that once again has a CSA geometry (Scheme 2N). During the entire process, the metal passes from +7 to +5 and *vice versa*. Once D binds the carbon forming *o*-CH_2_D the D does not participate anymore to the new agostic interaction because of the major strength of the C–D bond compared to the C–H one. The possible presence of a trihydrogen molecule involved in the process as postulated by several authors for complexes of: Ir,^43,45^ Mo,^46^ Re,^8^ and Fe,^47^ has been excluded by our DFT calculations, as we will see later in the article. Moreover, the pressure exerted by the mesityls of the NHCs pushes the η^2^-H_2_ continuously toward the less sterically hindered hydrides, and such equilibrium takes place so fast that it is out of the picoseconds-nanoseconds NMR timescale. In our case, the TTP or CSA geometry of 1–3, should be thought as a highly distorted one, and we may think that the distortion is part of the edge stretching needed to pass from one structure to the other one as in the reaction cycle developed by Muetterties and Guggenberger.^48^ Moreover, we know that the complexes studied in this work are neither hypo- nor hyperelectronic because they all possess a stable 18-electron valence shell configuration. It is worth noting that for the homoleptic complex [ReH_9_]^2−^ the Jahn–Teller distortions can be excluded due to its electron precise electronic configuration in which the energy gap between the highest HOMO and the lowest LUMO is large enough to prevent such distortions.^42,49^ It is noteworthy that the 16-electron-complex [ReH_6_]^3−^ containing a Re atom in the +3 OS, despite its hypoelectronic configuration, possesses a regular octahedral structure^50^ as well as the electron precise 18-electron complex [ReH_6_]^5−^ in which formally Re has an OS of +1.^49^

**Scheme 3:**
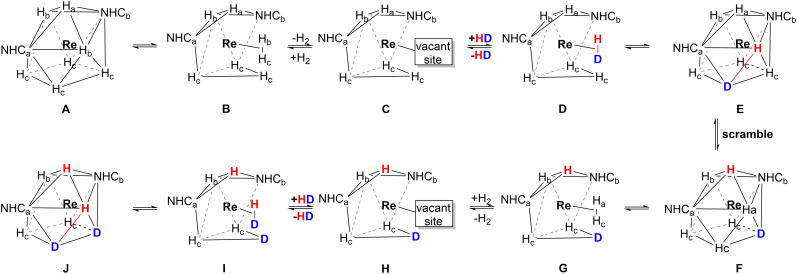
The fluxional mechanism involving the 2H ↔ η^2^-H_2_ rearrangement in complexes 1 and 3 at RT. This process leads to partial deuteration of 1 and 3. NHC ligands are omitted for clarity. At RT, C–H activation of the CH_3_ group on the mesityl ring is not feasible for 1 and 3. Consequently, the loss of H_2_ (steps C and H) creates a vacant site, which is subsequently occupied by coordination of an HD molecule (steps D and I). The continuous scrambling process results in mixing of D and H atoms around the Re center (steps F and L).

However, the fluxional mechanism we assign to 1 and 3, does not involve the formation of the *ortho* metalation as in the case of 2, but it still includes the formation of η^2^-H_2_ and its exchange with HD (Scheme 3). To gather further evidence for the formation of η^2^-H_2_ we conducted a hydrogenation experiment (Scheme S1) in which 1-hexene was hydrogenated to hexane (Fig. S6 and S7). The mechanism of such transformation implies the formation and subsequent loss of an η^2^-H_2_ molecule, and the formation of a vacant site where the substrate coordinates afterwards *via* its double bond (Scheme S1). It is worth noting that without the presence of η^2^-H_2_ the hydrogenation could not take place.

### Correlation between steric factors and the internal polyhydridic structure

Comparing the interbond angles of the known complex [ReH_7_(PPh_3_)_2_]^51^ with the ones of our complex 2^9^ we found that, for the complex bearing the phosphines, the angle P(1)–Re–P(2) is 138.9°(1) while the angle of the one bearing the carbenes C(1)–Re–C(22) is 154.63°(1). The difference between the angles is remarkable (Δ(°) = 15.73) and this is probably due to the higher buried volume that the IMes has when compared with PPh_3_. For instance, in a complex containing one coordinated IMes as in the case of [AuCl(IMes)] the buried volume (%*V*_bur_) reaches its maximum for this type of ligand (%*V*_bur_ = 36.5).^52^ Depending on the type and number of ligands bound to the metal the %*V*_bur_ of the IMes changes giving evidence of a certain flexibility of that NHC. When besides the NHCs, the ligands are hydrides, as in the case of 1–3, the %*V*_bur_ may reach a value (%*V*_bur_ = 35) really close to the maximum possible one. Furthermore, Perez-Acosta and co-workers, proved that the nature of the supporting ligands is responsible for the distribution of the hydrides around the metal centre and thus for the structure of the complex.^49^ In their work, the syntheses and characterization of Re polyhydride complexes bearing the diphosphine ligands were described: biphep (tropos biphenylphosphine), DPEphos (bis-[2-diphenyl phosphino phenyl]ether) and Xantphos (4,5-bis(diphenylphosphino)-9,9-dimethylxanthene) with different bite angles (bite angle range: *βn* ∼ 100–110°). The choice of hindered *cis* ligands had two main effects on the complex: a thermal instability as a consequence of the steric hindrance of the phosphorous ligands that caused a poor π-back-bonding toward the rest of the ligands, and a favoured intermolecular exchange of the hydrogens. A single phosphorus signal in the decoupled ^31^P-NMR confirmed the fluxionality of the hydrides for all three complexes abovementioned, while a coupled ^31^P-NMR gave an octet providing evidence of seven equivalent hydrides bound to Re. Considering previous results on similar complexes, and from the results obtained by means of variable temperature studies, the authors assigned to the Re(vii) polyhydrides bearing chelating bis-phosphine ligands a TTP geometry in which the phosphines occupy the eclipsed prism positions. A *T*_1 min_ of 61 ms at 233 K was found for the complex containing the DPEphos ligand, while for the other two complexes the authors were not able to obtain the *T*_1 min_ values because the solvent in which the complexes were dissolved froze before measurements could be completed. Crabtree and co-workers in a former work showed how different d^0^ polyhydrides reacted with HBF_4_ in acetonitrile to yield reduced solvento hydride complexes.^53^ For instance, the complex [(PPh_3_)_2_ReH_7_] analogous to our complex 3, reacted with HBF_4_ in MeCN, giving the reduced complex [(PPh_3_)_2_Re(iii)H(MeCN)_4_]^2+^ with an assigned pentagonal biprismatic geometry due to the bulkiness of the phosphines. The reaction of the complex [(PPh_3_)_2_ReH_7_] is in this case surprising since a protonation is not expected in a metal having an empty valence shell. This is why the authors supposed a reaction involving a three-centre intermediate system as Re⋯H⋯H^+^ that could be either linear or bent.

### DFT calculations

All theoretical calculations have been carried out with the ORCA 6 software package.^54^ Following the experimental evidence of the presence of η^2^-H_2_ and of the internal equilibrium [Re(vii)H_7_(NHC)_2_] ↔ [Re(v)H_5_(η^2^-H_2_)(NHC)_2_] for 1–3, we performed DFT calculations to gain more insights into both this equilibrium, and the possible polytopal rearrangements involving η^2^-H_2_. In these calculations, only fluxional mechanisms involving the hydrides were considered, while the NHCs of 1–3 were assumed not to participate in the polytopal processes because of their bulkiness. Calculations were run using the B3LYP functional with D3 dispersion corrections and the def2-SVP basis set, where the innermost 60 electrons of Re are replaced with an effective core potential. First, geometry optimizations were performed to identify the equilibrium structures of the two isomers [Re(vii)H_7_(NHC)_2_] and [Re(v)H_5_(η^2^-H_2_)(NHC)_2_]. For all the complexes 1–3, both optimized geometries were found to be local minima, as confirmed by analysing the eigenvalues of the Hessian matrix. The formation of η^2^-H_2_ is supported by the presence of a single H⋯H pair with an interatomic distance between 0.92 Å and 0.93 Å. The resulting energy difference between the dihydrogen and the dihydride forms, corresponding to the energy of formation of η^2^-H_2_ (Table 4), shows the lowest Δ*E*° value (−0.6 kcal mol^−1^) for 2, and the highest (+0.1 kcal mol^−1^) for 1.

**Scheme 4:**
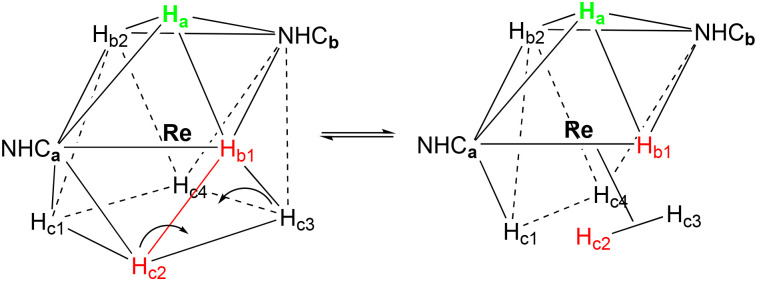
The 2H^−^ ↔ η^2^-H_2_ rearrangement for the process involving just two hydrides for complexes 1–3 in the CSA geometry.

This same qualitative order is reproduced at different levels of theory (Table 3). In order to compare the dynamics of the different fluxional mechanisms, we studied activation barriers and eventual reaction intermediates for several possible processes of H^−^ polytopal rearrangement. For each process, a minimum energy path was found by performing a nudged elastic band^55^ (NEB) optimization between the reactant and product structures. The resulting highest energy image was subsequently used as an initial guess for transition-state (TS) optimization; activation barriers were then estimated as the energy difference Δ*E*^#^ = *E*_TS_ − *E*_reac_. For the process of formation of η^2^-H_2_, a first minimum energy path (P1) was found. It involves only a simple approach for two of the H^−^ forming the square, up to an interatomic distance of 0.92 Å (Scheme 4). For all the complexes 1–3, the calculated energy barrier Δ*E*^#^ is very close to 1 kcal mol^−1^, indicating a very fast 2H^−^ ↔ η^2^-H_2_ interchange at RT.

**Table 3:** Values of Δ*E* for the process [ReH_7_(NHC)_2_] → [ReH_5_(η^2^-H_2_)(NHC)_2_] calculated using different DFT methods. B3LYP/def2-TVP energies are obtained from single-point calculation on-top of the B3LYP/def2-SVP relaxed geometries

NHC	B3LYP/def2-SVP	B3LYP/def2-TZVP	r^2^SCAN/def2-TZVP
IMes	0.1	1.5	1.2
BIMes	−0.5	1.0	0.7
IMesCl_2_	−0.6	0.8	0.5

**Table 4:** Δ*E* for the process of formation of η^2^-H_2_ for complexes 1–3 involving two hydrides. Energy in kcal mol^−1^ units

Process	1		2		3	
	Δ*E*°	Δ*E*^#^	Δ*E*°	Δ*E*^#^	Δ*E*°	Δ*E*^#^
P1	0.1	1.1	−0.6	0.8	−0.5	0.9

**Scheme 5:**
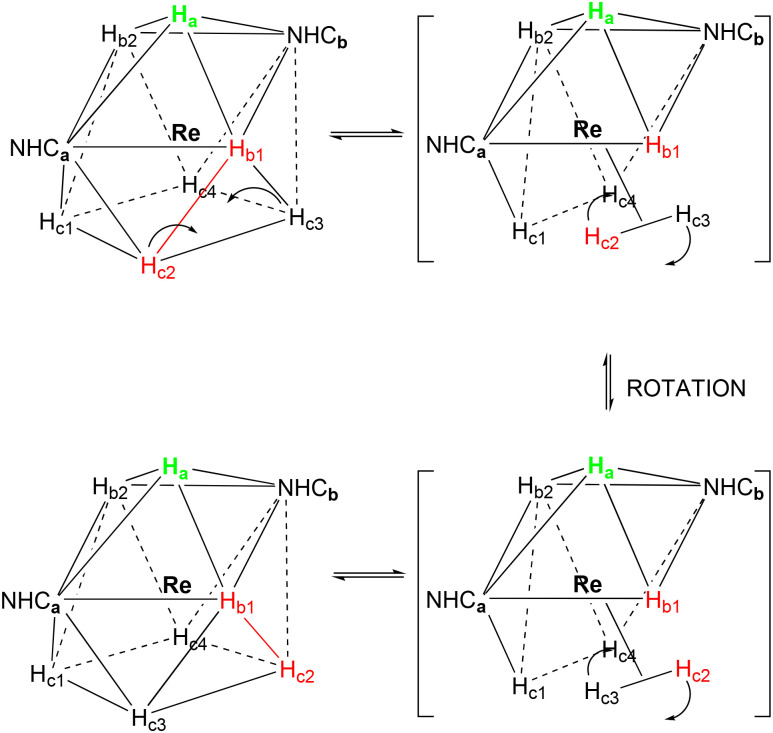
The 2H^−^ ↔ η^2^-H_2_ rearrangement for the process involving five hydrides for complexes 1–3 in the CSA geometry.

Additionally, a second process that can lead to the same η^2^-H_2_ product through a different TS was identified. This alternative process (P2) involves a concerted reorganisation of five hydrides: the four H^−^ forming the square of the CSA, and one of the three H^−^ that forms the pyramid together with the NHCs (Scheme 5). The five H^−^ involved dispose themselves in way to have the higher distance from each other.

In this case, the Δ*E*^#^ of the process is larger for all three complexes compared to the previous mechanism (Table 5), although the values of 2.1–2.5 kcal mol^−1^ are still small enough to allow for a very fast interconversion dynamics.

**Table 5:** Δ*E* for the process of formation of η^2^-H_2_ for complexes 1–3 involving five hydrides

Process	1		2		3	
	Δ*E*°	Δ*E*^#^	Δ*E*°	Δ*E*^#^	Δ*E*°	Δ*E*^#^
P2	0.1	2.5	−0.6	2.5	−0.5	2.1

We then focused on those mechanisms where the polytopal rearrangement only results in an interchange of H^−^ positions between the sites of the CSA geometry, with the reactant and product being fluxional isomers of identical energy. In the first isoergic processes that we considered (P3), a η^2^-H_2_ intermediate is initially formed *via* either P1 or P2, followed by the reverse of the other mechanism to restore 2H^−^. This overall process has the same Δ*E*^#^ as P2 (Table 6), which exhibits higher barriers than P1 and therefore, results to be the rate-limiting step. This means that, thanks to the very low activation energies, all three proposed mechanisms P1–P3 could be easily responsible for the internal scrambling of the hydrides.

**Table 6:** Δ*E* of the H-scramble for complexes 1–3 involving a mix of both P1 and P2 processes

Process	1	2	3
	Δ*E*^#^	Δ*E*^#^	Δ*E*^#^
P3	2.5	2.5	2.1

**Scheme 6:**
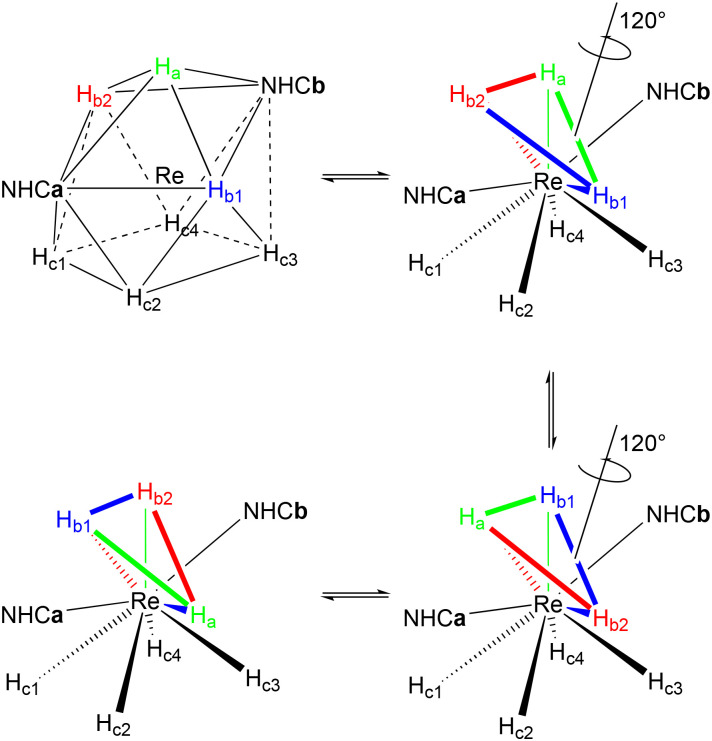
The turnstile mechanism for complexes 1–3 in the CSA geometry.

The turnstile mechanism proposed by Crabtree^56^ is another fluxional mechanism that we analysed for the complexes 1–3. The triangle formed by the three hydrides: H_a_ and the two H_b_, is subject to a 120° turnstile rotation motion about the Re. This mechanism may also occur with the triangle formed by H_b1_, H_c2_ and H_c3_ below it, or by H_b2_, H_c1_ and H_c4_ below the latter (Scheme 6). For our calculations, we considered that this mechanism takes place through a transition state involving an intermediate rotation of 60° (Table 7).

**Table 7:** Δ*E* for the turnstile process for complexes 1–3

Process	1	2	3
	Δ*E*^#^	Δ*E*^#^	Δ*E*^#^
Turnstile	10.6	9.4	9.8

**Scheme 7:**
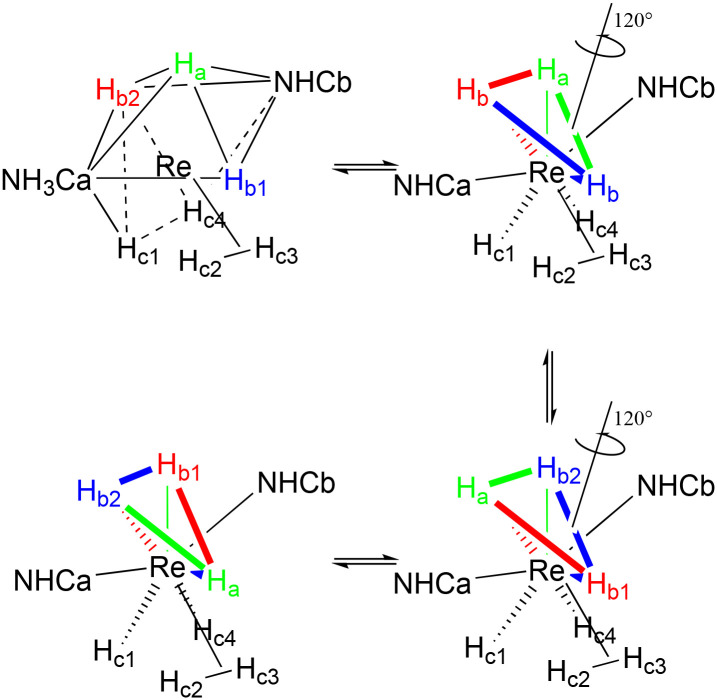
The η^2^-turnstile mechanism for complexes 1–3.

For the turnstile mechanism, the difference in enthalpies is higher than the one found for the processes P1–P3. However, the Δ*E*^#^ values are still in a range that makes it a possible polytopal rearrangement. A special case of the turnstile mechanism is the one in which the turnstile takes place in the presence of η^2^-H_2_ (η^2^-turnstile) (Scheme 7). For this process we found a slightly lower Δ*E* value for 3 (Table 8), but still in the same range of the conventional turnstile.

**Table 8:** Δ*E* for the η^2^-turnstile process for complexes 1–3

Process	1	2	3
	Δ*E*^#^	Δ*E*^#^	Δ*E*^#^
η^2^-Turnstile	10.3	10.0	8.8

The values of Δ*E* for the 180° rotation of η^2^-H_2_ around the Re-η^2^-H_2_ axis are quite high when compared with the Δ*E* for the formation of the η^2^-H_2_, however are low enough to make the process possible (Table 9).

**Table 9:** Δ*E* for the rotation of η^2^-H_2_ around the Re-η^2^-H_2_ axis for complexes 1–3

Process	1	2	3
	Δ*E*^#^	Δ*E*^#^	Δ*E*^#^
η^2^-H_2_	8.7	8.0	7.5

The analysis of the quadratic twist process for 1–3 showed us that it does not occur directly through a single 90° rotation of the four H^−^ that form the base of the CSA, since the TS, with an intermediate rotation of 45°, would require a very high energy (close to 40 kcal). The same result of the quadratic twist is instead reached, with much less energy, passing through a process that involves an intermediate species that imply the presence of η^2^-H_2_ (η^2^-quadratic twist), and a rotation of the three H^−^ belonging to the starting square of the CSA (Table 10).

**Table 10:** Δ*E* for the η^2^-quadratic twist for complexes 1–3

Process	1	2	3
	Δ*E*^#^	Δ*E*^#^	Δ*E*^#^
η^2^-Quadratic twist	8.8	7.4	7.0

**Scheme 8:**
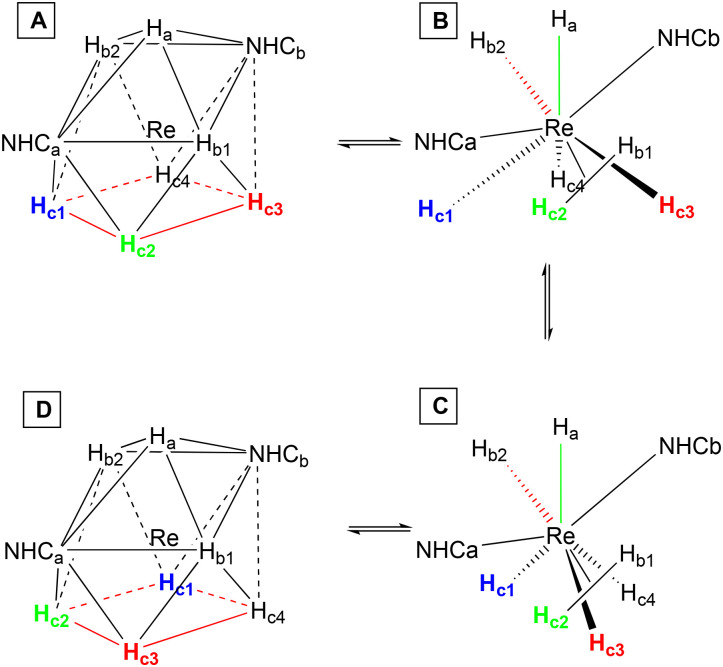
The dynamics of the η^2^-quadratic twist process involving the presence of η^2^-H_2_ for complexes 1–3.

The result of the η^2^-quadratic twist is equivalent to the 90° rotation of the four hydrogens involved; however, the final configuration is reached by passing through the intermediate [Re(H)_5_η^2^-H_2_(NHC)_2_]. The overall process can be divided into three steps: the formation of the [Re(H)_5_η^2^-H_2_(NHC)_2_] isomer (B), the rotation of the three H^−^ that belong to the starting square of the CSA through a 90° rotation (C), and finally the return to the [Re(H)_7_(NHC)_2_] configuration (D) (Scheme 8).

**Fig. 5:**
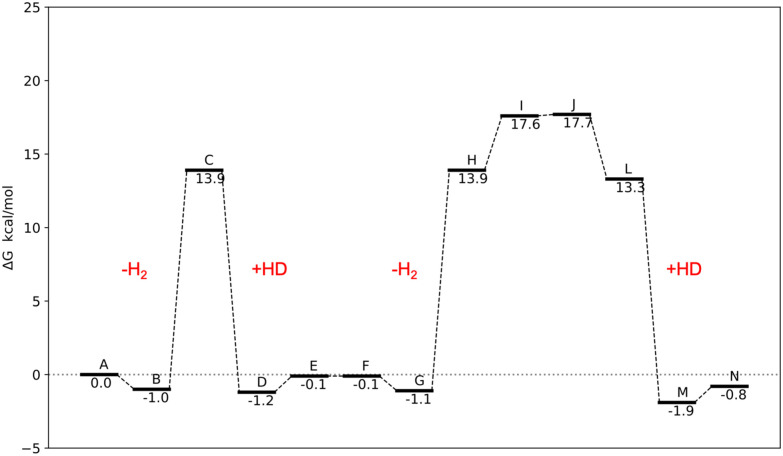
Reaction free energies (*T* = 293 K) for the steps of the mechanism proposed in the Scheme 2. Step K has not been included since the complex shows the same configuration of the step J.

**Fig. 6:**
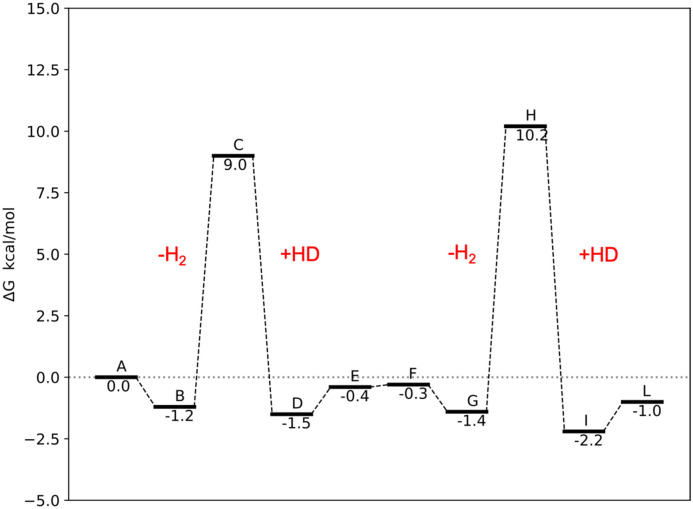
Reaction free energies (*T* = 293 K) for the steps of the mechanism proposed in the Scheme 3.

**Fig. 7:**
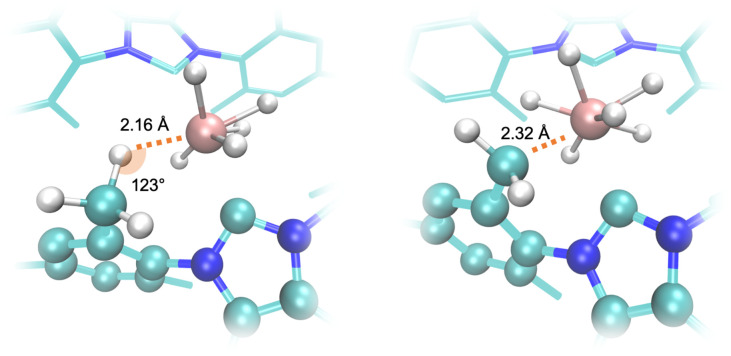
Geometric details of the optimized structures of 2 in Scheme 2. Left: the agostic C–H–Re interaction (step C). Right: the process of breaking the C–H bond of a *o*-CH_3_ in a mesityl of IMesCl_2_ and the subsequent formation of a C–Re bond.

To evaluate the proposed mechanisms of the H/D exchange (Schemes 2 and 3), we performed the DFT calculations of the Gibbs free energies of the reaction, which include both vibrational energy and entropy to account for the effect of the H–D isotopic substitution. We started calculating the energy profile of the transformation that leads from 2 (Scheme 2A) to the corresponding doubly-deuterated product (Scheme 2N) (Fig. 5). The exchange of η^2^-H_2_ (or HD) molecules is predicted to be strongly endothermic, with Δ*G* above 17 kcal mol^−1^. It is worth noting that these values do not consider effects of the environment, *e.g.* differential solvation of the reactant and the products, which may significantly alter the energetic landscape of an AB → A + B type of reaction. The predicted values are high for reactions at RT, and this accounts for the long reaction time (72 h), high temperature (60 °C), and pressure (2 atm of HD), needed to bring the reaction to completion.^9^ However, the calculations show that both the predicted agostic interaction (Scheme 2C, H) and the *ortho*-metalation (Scheme 2I), are sufficiently strong to lead to the formation of stable intermediates once the η^2^-H_2_ has left the complex. The interatomic distance and angle in the optimized geometry of these intermediates are shown in Fig. 7. Conversely, as anticipated before, no trihydrogen intermediate has been found neither as an energy minimum nor as a TS. The alternative H/D exchange mechanism without *ortho*-metalation (Scheme 3) was also considered. Compared to the previous mechanism, the calculated Δ*G* values for 2 (Fig. 6) show that this process needs significantly less energy in order to proceed. This is due to the fact that the pentahydride complex with a vacant site (Scheme 3C), which forms after the loss of η^2^-H_2_, is predicted to be more stable than the C–H–Re agostic interaction and the C–Re bond by about 4 and 7 kcal mol^−1^, respectively. Accordingly, the subsequent deuteration of hydrides in the ReH_7_ moiety is expected to occur faster than the H/D exchange involving a *o*-CH_3_ group of the mesityl substituent of the NHCs.

## Conclusions

In this work we proposed fluxional mechanisms that may occur inside the Re nona-coordinated complexes 1, 2 and 3 in their CSA distorted geometry. We provided evidence that in Re polyhydride complexes the steric hindrance of the ligands can influence the equilibrium 2H^−^ ↔ η^2^-H_2_ towards the Re(v) species with a molecule of H_2_ coordinated η^2^. Through DFT calculations, we were able to find two new polytopal rearrangements: η^2^-turnstile and η^2^-quadratic twist. For 2 we suggested a fluxional mechanism that considers its internal *ortho* metalation. The latter process does not occur in 1 and 3, for which we suggest a different mechanism that in any case considers the presence of η^2^-H_2_. Furthermore, the presence of the latter inside all three complexes has been shown by the labelling experiments of 1–3, and a catalytic hydrogenation experiment in which 2 has been used as a catalyst.

## Author contributions

GG conceived the project, wrote the original draft, reviewed and edited it, synthesized the catalyst used for the experiments and conducted both experiments and NMR analysis. AP performed the DFT calculations. MP supervised the DFT calculations. All three authors contributed in drawing the conclusions.

## Conflicts of interest

There are no conflicts to declare.

## Supplementary Material

DT-054-D5DT01655J-s001

## Data Availability

The data supporting this article have been included in both the supplementary information of this article, and in the supplementary information of ref. 9. Supplementary information: Scheme S1, Experiment procedure, Fig. S1 and S2. See DOI: https://doi.org/10.1039/d5dt01655j
