# Supplementary material for: Dynamic processes of the first NHC-substituted rhenium heptahydrides [ReH7(NHC)2]
Source: Dalton Trans. 2025 Aug 4;54(35):13246–56. doi: 10.1039/d5dt01655j (PMC12678046; doi:10.1039/d5dt01655j)
Supplement: DT-054-D5DT01655J-s001 [file DT-054-D5DT01655J-s001.pdf]

Supplementary Information (SI) for Dalton Transactions.
This journal is © The Royal Society of Chemistry 2025

Supporting Information

Dynamic processes of the First NHC-Substituted Rhenium Heptahydrides [$ReH_7(NHC)_2$]

Gabriele Grieco[a]*, Adriano Pierini[b], Marco Pierini[c]

a University of Zurich, Department of Chemistry, Winterthurerstrasse 190, CH-8057 Zurich, Switzerland.

*E-mail: pgag.2021@gmail.com, Fax: +41 44 635 68 02

b Sapienza University of Rome, Department of Chemistry, Piazzale Aldo Moro, 5, 00185 Roma RM, Italy.

E-mail: adriano.pierini@uniroma1.it

c Sapienza University of Rome, Department of Drug Chemistry and Technology, Piazzale Aldo Moro, 5, 00185 Roma RM, Italy.

Table of contents

Scheme S1 **3**

Experiment procedure **3**

Figure S1 **4**

Figure S2 **4**

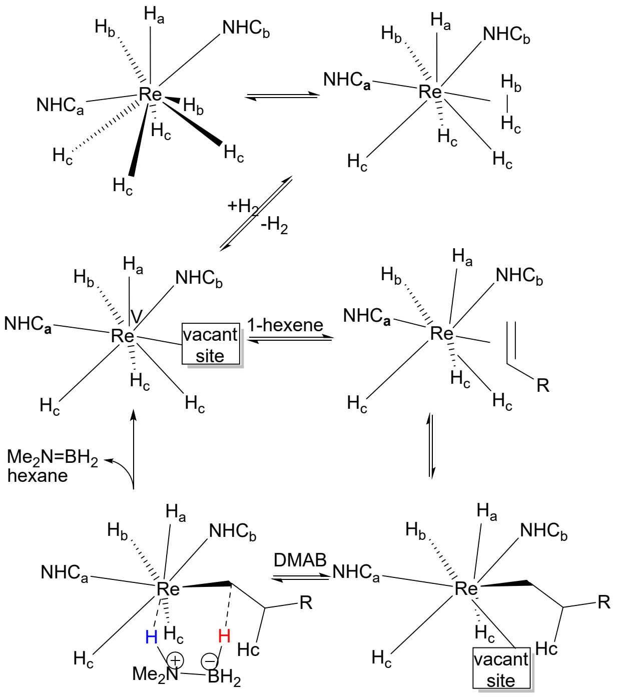

Scheme S1. Catalytic cycle implying the formation and loss of η^2-H_2 for the complex **1**. Both NHC_a and NHC_b are IMes.

Experiment used to give further evidence of the presence of η^2-H^2 in 1

Catalyst **1** (0.0012 mmol, 1 mg), 1-hexene (0.31 mmol, 45 μL) and borane dimethylamine complex (DMAB) (0.017 mmol, 1 mg) were placed in an NMR tube with a Teflon screw cap, and then dissolved in THF-d_8 (0.5 mL). After 20 minutes the ^{1}H-NMR showed both 2-hexene (isomerization) and hexane, together with the starting material (Figure S1). After 60 minutes at 70°C the ^{1}H-NMR analysis showed the complete hydrogenation of the substrate (Figure S2).

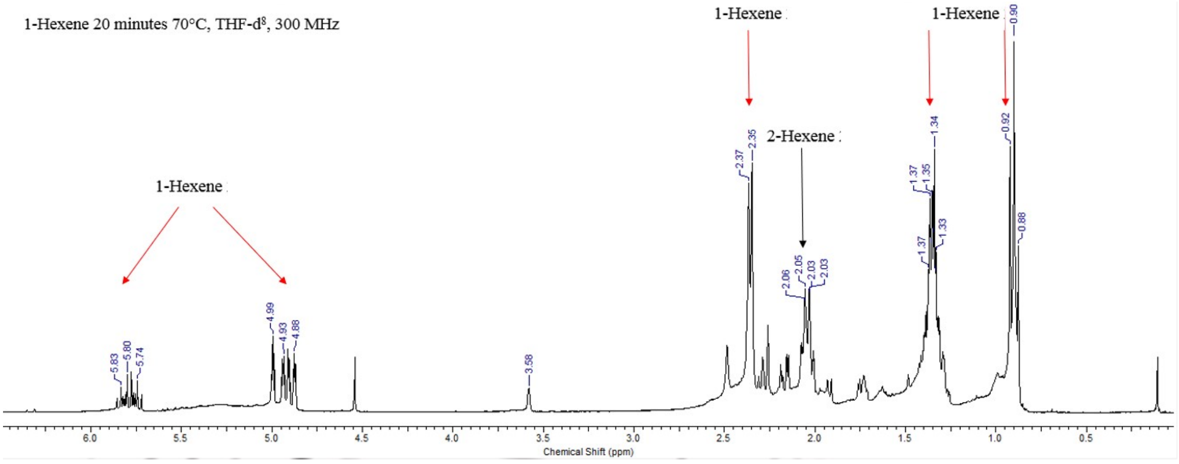

Figure S1. ^{1}H-NMR spectra of the hydrogenation of 1-hexene after 20 minutes at 70°C (THF-d_8, 300 MHz). Three species are present: starting material, 2-hexene and the product (hexane).

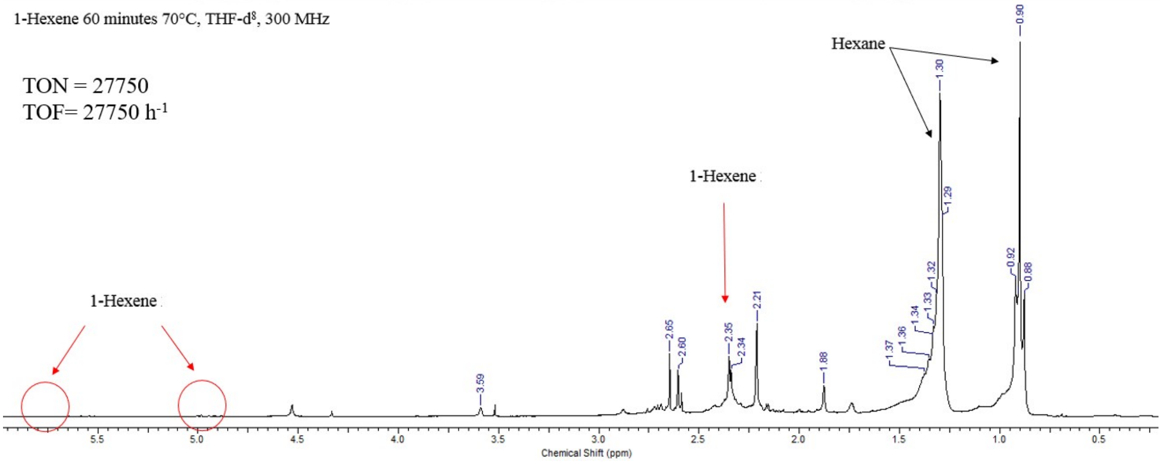

Figure S2. ^{1}H-NMR spectra of the hydrogenation of 1-hexene after 60 minutes at 70°C (THF-d_8, 300 MHz). Traces of the starting material (1-hexene) are present together with the product (hexane).

Note after first publication

This Supplementary Information document replaces the version originally published on 4th August 2025, which included NMR spectra reproduced from a previous publication (these are now referenced and discussed in the text of the article instead).
